# An Interactive Physical-Cognitive Game-Based Training System Using Kinect for Older Adults: Development and Usability Study

**DOI:** 10.2196/27848

**Published:** 2021-10-27

**Authors:** Teerawat Kamnardsiri, Kochaphan Phirom, Sirinun Boripuntakul, Somporn Sungkarat

**Affiliations:** 1 Research Group of Modern Management and Information Technology College of Arts, Media and Technology Chiang Mai University Chiang Mai Thailand; 2 Department of Digital Game College of Arts, Media and Technology Chiang Mai University Chiang Mai Thailand; 3 Department of Physical Therapy Faculty of Associated Medical Sciences Chiang Mai University Chiang Mai Thailand

**Keywords:** digital game, interactive game-based training, physical-cognitive training, exergaming, Kinect sensors, older adults, falls, PACES, user-centered design, game-based exercise

## Abstract

**Background:**

Declines in physical and cognitive functions are recognized as important risk factors for falls in older adults. Promising evidence suggests that interactive game-based systems that allow simultaneous physical and cognitive exercise are a potential approach to enhance exercise adherence and reduce fall risk in older adults. However, a limited number of studies have reported the development of a combined physical-cognitive game-based training system for fall risk reduction in older adults.

**Objective:**

The aim of this study is to develop and evaluate the usability of an interactive physical-cognitive game-based training system (game-based exercise) for older adults.

**Methods:**

In the development phase (Part I), a game-based exercise prototype was created by integrating knowledge and a literature review as well as brainstorming with experts on effective fall prevention exercise for older adults. The output was a game-based exercise prototype that covers crucial physical and cognitive components related to falls. In the usability testing (Part II), 5 games (ie, Fruits Hunter, Where Am I?, Whack a Mole, Sky Falls, and Crossing Poison River) with three difficulty levels (ie, beginner, intermediate, and advanced levels) were tested in 5 older adults (mean age 70.40 years, SD 5.41 years). After completing the games, participants rated their enjoyment level while engaging with the games using the Physical Activity Enjoyment Scale (PACES) and commented on the games. Descriptive statistics were used to describe the participants’ characteristics and PACES scores.

**Results:**

The results showed that the average PACES score was 123 out of 126 points overall and between 6.66 and 7.00 for each item, indicating a high level of enjoyment. Positive feedback, such as praise for the well-designed interactions and user-friendly interfaces, was also provided.

**Conclusions:**

These findings suggest that it is promising to implement an interactive, physical-cognitive game-based exercise in older adults. The effectiveness of a game-based exercise program for fall risk reduction has yet to be determined.

## Introduction

Declines in multiple physiological systems with ageing contribute to balance and gait deficits, leading to an increased risk of fall [1]. Fall is a serious public health problem, and its consequences have a marked adverse impact on physical and psychological aspects such as injuries, activity restriction, fear of falling, and loss of autonomy [2-4]. Given the substantial impact of falls on health, as well as their medical and economic burden, an effective strategy to prevent falls in older adults is warranted.

Several investigators have consistently reported a strong positive effect of physical exercise on fall prevention among older adults [5-9]. Researchers have also identified a critical role of cognition, especially executive function, attention, and memory, on balance and gait control [10-13]. Many examiners have demonstrated that cognitive training, which is an intervention program aimed at improving, maintaining, or restoring cognitive function via the repeated and structured practice of tasks, can improve balance and gait and reduce fall risk [14-17]. Taken together, incorporating a cognitive component into physical exercise may augment its benefits in fall prevention [11-13,18-20]. A growing number of investigators have documented the effects of combined physical-cognitive exercise training in a simultaneous form (dual-tasking) among older adults. Combined physical-cognitive exercise training programs have resulted in greater improvement in physical and cognitive performance than either type of single training alone [21-25].

As technology advances, a new alternative of rehabilitation approach targeting training of various physical and cognitive components in the form of interactive game-based exercises (exergames) is becoming available [26]. These interactive game-based exercises use technology-driven platforms that require users to move their body in order to complete assigned tasks via video game interface elements [27]. The interactive game-based exercises have advantages in terms of gamification features. Researchers have demonstrated that exergames are attractive because they provide real-time interaction and feedback to users, which enhances motivation and training adherence [28,29]. In addition, game-based exercises allow users to be active with repetitive practice and track progression, which is beneficial for training outcomes [30]. Another advantage of exergames is that they can offer an experience for daily-life task requirements based on concurrent training of physical and cognitive components [31-33]. Moreover, game-based exercises can be applied either in rehabilitation centers or community and home settings [33,34]. Several researchers have shown that game-based training using Nintendo Wii Fit, and Microsoft Kinect sensors were effective in improving physical abilities (eg, balance, gait performance), improving cognitive abilities (eg, executive function, speed of processing), and reducing the risk of falls among older adults [35-42]. However, in most existing training programs available for fall prevention, research concerning simultaneous training of physical and cognitive functions (dual tasking) with the use of exergames has remained scarce.

Among interactive game-based technology for training, the Kinect motion sensor (Microsoft Corporation) has been considered as a high-potential approach because it provides a markerless full-body 3D motion tracker and enables users to virtually interact hands-free with a computer system. Additionally, several examiners have demonstrated that among interactive game-based technology for exercising, Microsoft Kinect has an advantage in that it allows individuals to interact with games using their own body in a natural way [43,44]; this enhances the natural form of human-computer interaction [45]. In addition, the Microsoft Kinect motion sensor is an accurate input device; thus, it allows precise tracking and real-time feedback of user performance [46]. The Microsoft Kinect sensor is proposed to be a feasible and effective tool for training concurrent physical and cognitive components in older adults, with the aim of reducing the intrinsic causes (ie, physical and cognitive) of falls [47-51].

Based on the usability challenges faced by older adults, programmers should develop user interfaces that are user-friendly for older adults and specific for training purposes. A previous investigator has suggested that older adults accept innovative technology when they recognize its benefit and find it meaningful for their lives [52].

Therefore, this study focused on developing a prototype of a game-based exercise that accounts for the target user’s expectation and requirement (eg, enjoyment, attractiveness, user skill-challenge balance, and benefit for training cognitive and physical components). In particular, following a user-centered design approach, acceptability and training adherence in future empirical studies were established.

## Methods

### Study Design

**Figure 1:**
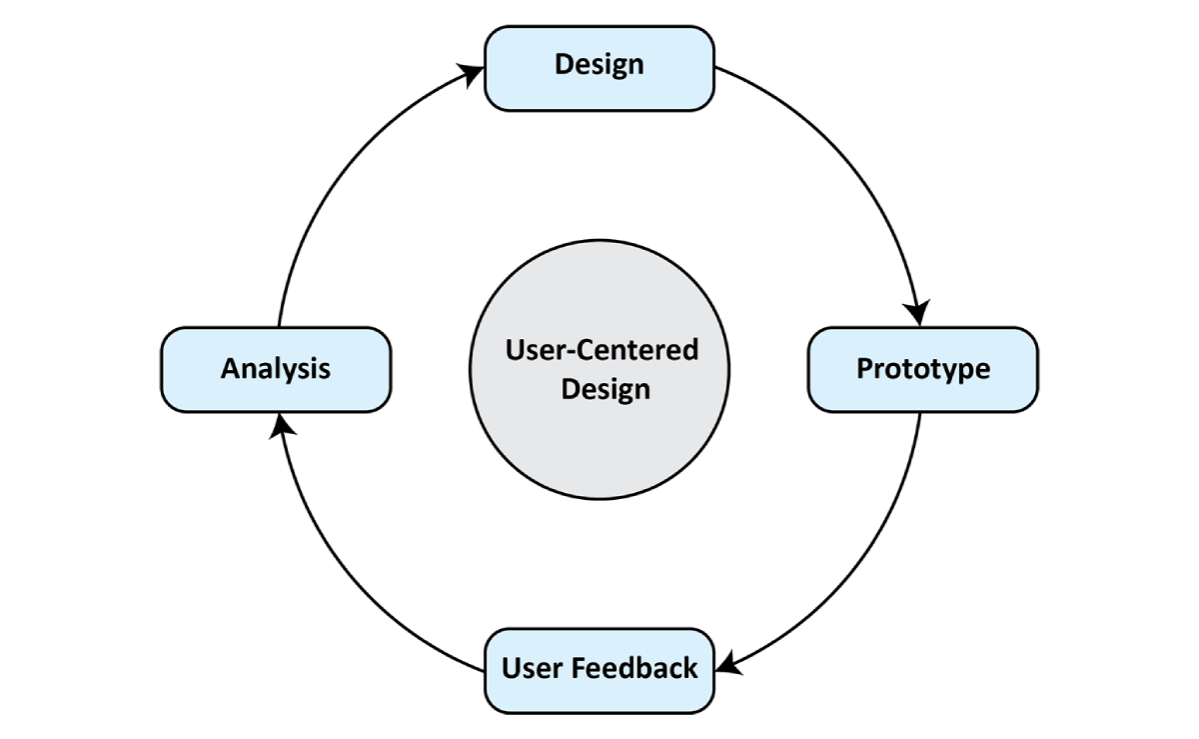
Schematic of the user-centered design cycle [27].

The present study consists of two parts: (1) the development of a game-based exercise prototype, and (2) evaluation of target users’ feedback, response, and satisfaction regarding the developed game-based exercise prototype. The concept of development and evaluation of the target user’s experience for producing a final prototype followed a user-centered design (UCD) approach [27,46,53-62]. UCD is a user interface design process in which designers focus on gaining a target user’s perspective to create a product with a high degree of usability. The UCD cycle is depicted in Figure 1.

### Part I: Development of a Game-Based Exercise Prototype

#### Development Process

In this part, the 4-phase UCD process was applied [27,46,53-62]. The first phase, the design development process of a game-based exercise prototype, was conducted in the brainstorming phase. A total of 7 team members, including 3 physical therapists and 1 physician (3-20 years of experience in geriatric and cognitive rehabilitation), 2 game programmers (5 years of experience in the Unity 3D game engine), and 1 game designer (10 years of experience in game design and game theory), participated in the brainstorming session. This phase involved generating potential core game ideas by integrating the knowledge and literature review of previous physical and cognitive training programs and interactive exergame interventions for fall prevention in older adults [16,63-65]. In the present study, the core game was composed of two training elements: (1) a physical element, including stepping and balance training, and (2) a cognitive element related to balance and falls in older adults, including executive function, attention, and memory [18,66,67]. To ensure that the difficulty of the games was appropriate for each individual user, the progression of game difficulty was considered. The game-based training program underwent critical appraisal by the physical therapists and physician. In the second phase, after consensus, proven game ideas were used to create a game-based digital exercise game prototype using the Unity 3D game engine software with Kinect Sensor V2 for Windows. In the third phase, user feedback to improve the game-based exercise prototype was provided by end users using a think-aloud method [68]. Finally, the fourth phase concentrated on assessing physical activity enjoyment during game engagement. The Physical Activity Enjoyment Scale (PACES) questionnaire [69,70], an 18-item scale questionnaire, was used to analyze physical activity enjoyment. Moreover, the usability of the game-based exercise prototype was determined with a structured interview (feedback about the game, themes, user interface, sound effect, graphics, and interaction). Feedback from all participants was considered by the research team to improve the game-based exercise prototype.

#### Characteristics of the Game-Based Exercise Prototype

The characteristics of the game-based exercise prototype are presented in Table 1. The game can be described as individual interactive game-based training using Kinect. The prototype of the game-based exercise comprised 5 games, including (1) Fruits Hunter, (2) Where Am I ?, (3) Whack a Mole, (4) Sky Falls, and (5) Crossing Poison River. The games had three different levels: beginner, intermediate, and advanced. The level of game complexity progressed by increasing the difficulty of physical demand (ie, movement speed, distance, duration, base of support) and cognitive demand (ie, number of stimuli, complexity of the game’s rules, and amount of cognitive load). The estimated play time was 45 to 60 minutes.

**Table 1:** Summary of the characteristics of the developed game-based exercise prototype.

Characteristic				Description
**Basic characteristics**				
	Health topic			A game-based exercise prototype
	Targeted age group			Older adults (age ≥65 years)
	Short description of the game idea			The game-based exercise is a virtual, interactive game-based training system using Microsoft Kinect motion sensor technology. The game-based exercise comprises 5 games that include physical and cognitive components associated with balance and falls in older adults.
	Target player			Individual
	Behavior change procedure used			A game-based exercise is used to enhance motivation and engagement in older adults.
	Clinical support needed			Physical therapist and geriatric physicians
	Data shared with clinician			Data are saved and stored in the hard disk. However, reaction time, error, and score are given as feedback on the display screen (ie, the rubber mat) at the end of each game.
	Type of game			Physical, action, real-time strategy
**Game components**				
	**Player’s game goal/objective**			
		Physical components	Improve static and dynamic balance Improve stepping reaction and response time Improve lower limb muscle strength	
		Cognitive components	Fruits Hunter: improves response ability and speed of processing via a stepping task. Where am I ?: improves semantic memory and visuospatial ability via visual sense Whack a Mole: improves selective attention ability, visual attention performance, speed of processing, and inhibition ability Sky Falls: improves sequencing and planning ability Crossing Poison River: improve episodic memory via auditory sense	
		Rules	Fruits Hunter: step on the presented fruits as fast as possible within a limited time. Where am I ?: step to the presented objects and remember as many as of them possible. The recall questions are provided at the end of the game. Whack a Mole: respond correctly to different rules of this game as follows: Mole or rabbit: steps on the target 1 time Mole or rabbit with helmet: steps on the target 2 times Bomb: do not step on the target Sky Falls: step with alternating feet to collect as many dropping objects in the basket as possible Crossing Poison River: listen to a short story and remember the content of the story while standing on one leg	
	Game mechanics			The game-based exercise system allows users to interact with the virtual games by stepping on the presented targets in different directions in pursuit of the game’s goals. The game-based exercise also provides audio and visual feedback to the users while they are playing the games.
	Virtual environment			A forest with fruits, animals, vegetables, and a river
	Setting			The game-based exercise can be set in a room environment
	Device requirements			Personal computer/notebook/laptop with LED projector
	Sensors used			Microsoft Kinect Sensor V2
	Estimated play time			45-60 minutes

### Part II: Evaluation of Target User’s Experience

#### Recruitment and Participants

A total of 5 community-dwelling older adults were enrolled as representative target users. The inclusion criteria were (1) age 65 years or older, (2) had normal cognitive function (determined by a Mental State Examination T10 [71] score ≥24 points or depending on the level of education), (3) ability to walk without an assistive device for at least 10 m, and (4) ability to step in all directions independently and safely. Exclusion criteria were (1) depressive symptoms (determined by a Thai Geriatric Depression Scale-15 [72] score >6 points), (2) orthopedic deficits, neurological deficits, and/or other significant health problems that precluded the participant from completing the testing protocol, and (3) uncorrected visual and hearing impairment. The study protocol was approved by the Human Ethical Review Board of the principal investigator’s institute (AMSEC-61EX-078). All participants gave written informed consent prior to participating in the study. The demographic data of the participants, which consisted of age, height, weight, medication use, and history of falls in the previous 12 months, were recorded.

#### Hardware Configuration

**Figure 2:**
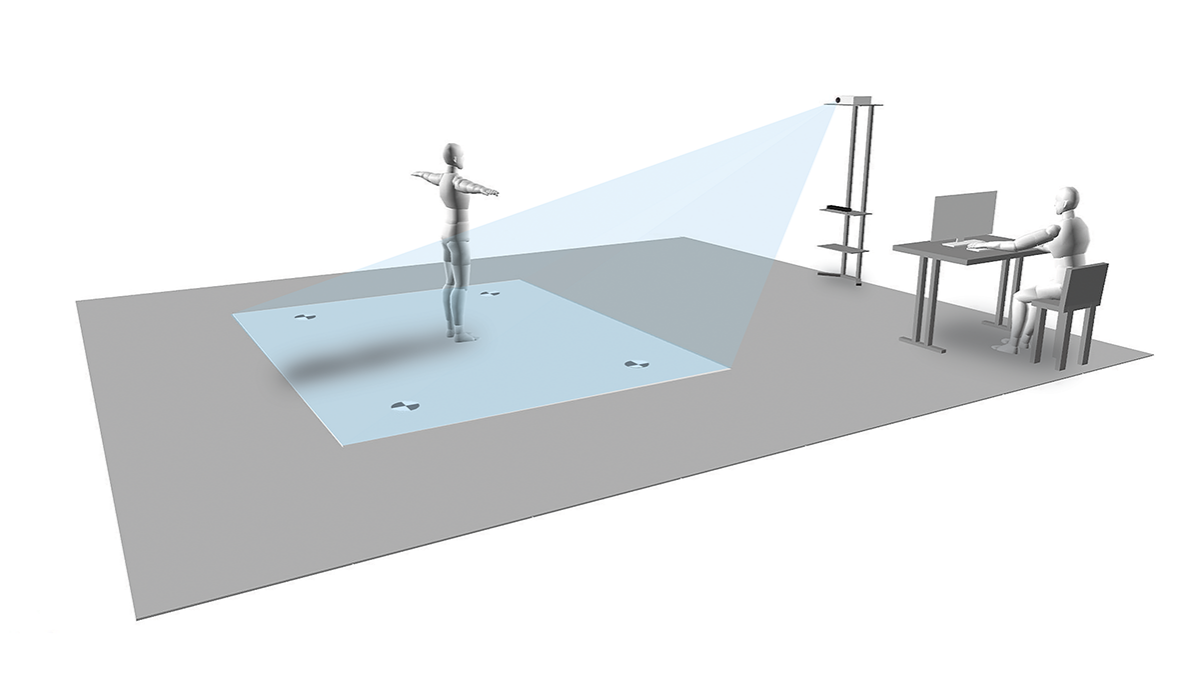
Environment configuration of the game-based exercise system.

To set up the system, the capture volume of the system was configured using the three main devices: Kinect Sensor V2 [73], LED projector, and laptop computer. In the present study, the Kinect Sensor V2 was used because it provides greater precision and more stable results compared to the Kinect Sensor V1 [74]. The Kinect Sensor V2 is a depth sensor camera manufactured by Microsoft that provides information about the depth, color, and skeleton of a user who is standing in front of the sensor. The Kinect sensor and LED projector were set on a portable metal storage rack at a height of 0.8 m and 2.0 m from the floor, respectively. The laptop computer that contained the developed game software was set near the Kinect sensor. The game was projected on a rubber mat (2.0 m width × 1.2 m height) that was placed on the floor; thus, the participants could virtually interact by stepping. The center of the rubber mat was set 2.5 m from the Microsoft Kinect sensor and LED projector. The configuration of hardware for playing the game-based exercise is illustrated in Figure 2.

#### Protocol

The game-based exercise was connected with the Microsoft Kinect sensor, an LED projector, and the laptop computer. After that, the system was calibrated by moving four markers in the game-based training system over the four corner marks on the rubber mat. For individualized body position calibration, each participant was asked to perform a T-pose stand for approximately 5 seconds at the center of the rubber mat (Figure 2). After the calibration process, each participant received a comprehensive description of the rules of the games, including a demonstration, and was requested to play the game-based exercise. The American College Sport of Medicine exercise guidelines recommend that older adults should participate in aerobic activities for a minimum of 30 minutes per session to promote and maintain their health-related outcomes [75]. In this study, the exercise duration ranged between 30 and 50 minutes, with a rest interval of between 10 and 15 minutes, depending on the participant performance; this resulted in a total time of 45 to 60 minutes.

After completing the game-based exercise, participants were asked to rate their enjoyment using the PACES questionnaire [69,70]. The PACES is an 18-item scale questionnaire that assesses physical activity enjoyment during game engagement with a 7-point Likert scale (1, strongly disagree, to 7, strongly agree). A higher PACES score reflects a greater level of enjoyment. Moreover, using a structured interview, participants were interviewed about their impressions of the game-based exercise features in terms of rules, mechanics, interfaces, and scoring, as well as their physical and cognitive involvement while playing the games.

#### Data Analysis

Descriptive statistics were used to describe both the participants’ characteristics and their scores on the 18-item PACES questionnaire. All data were analyzed using SPSS 21.0 (IBM Corporation).

## Results

### Part I: Development of a Game-Based Exercise Prototype

**Figure 3:**
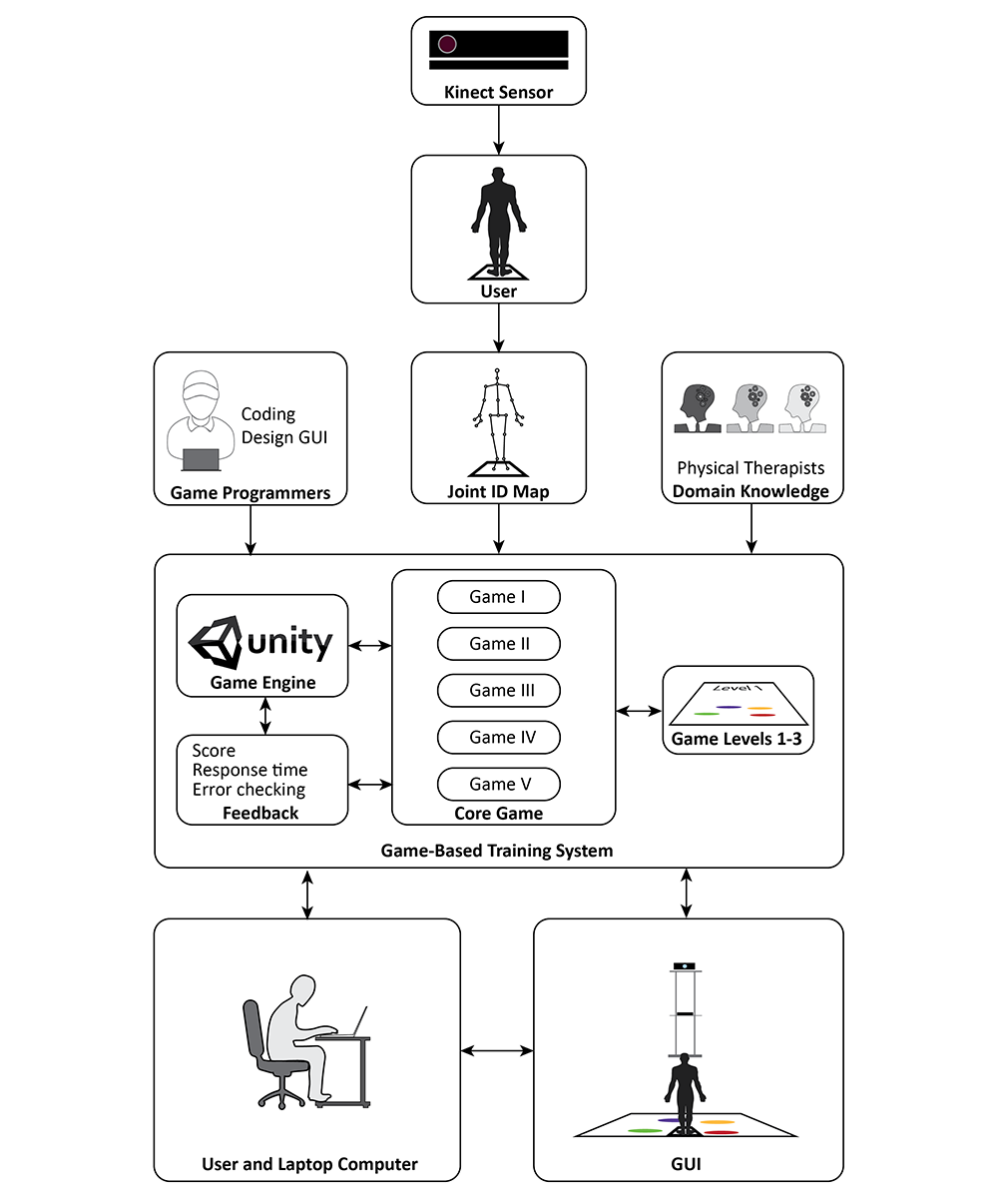
Framework of the game-based exercise system. GUI: graphical user interface.

**Figure 4:**
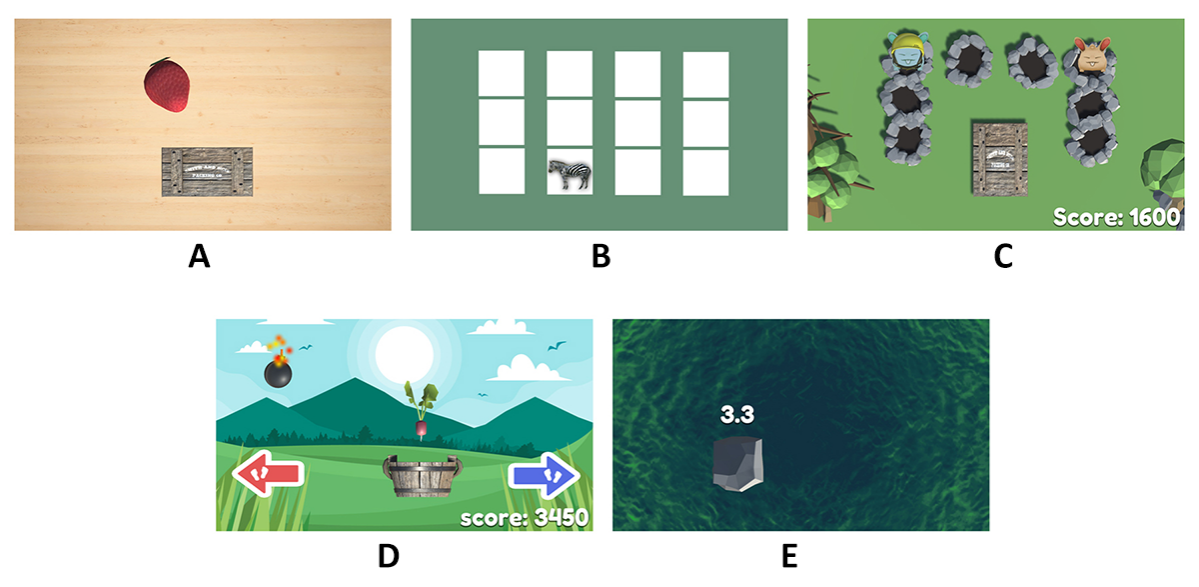
Screenshots of the 5 games in the game-based exercise system: (A) Fruits Hunter, (B) Where Am I ?, (C) Whack a Mole, (D) Sky Falls, and (E) Crossing Poison River.

**Figure 5:**
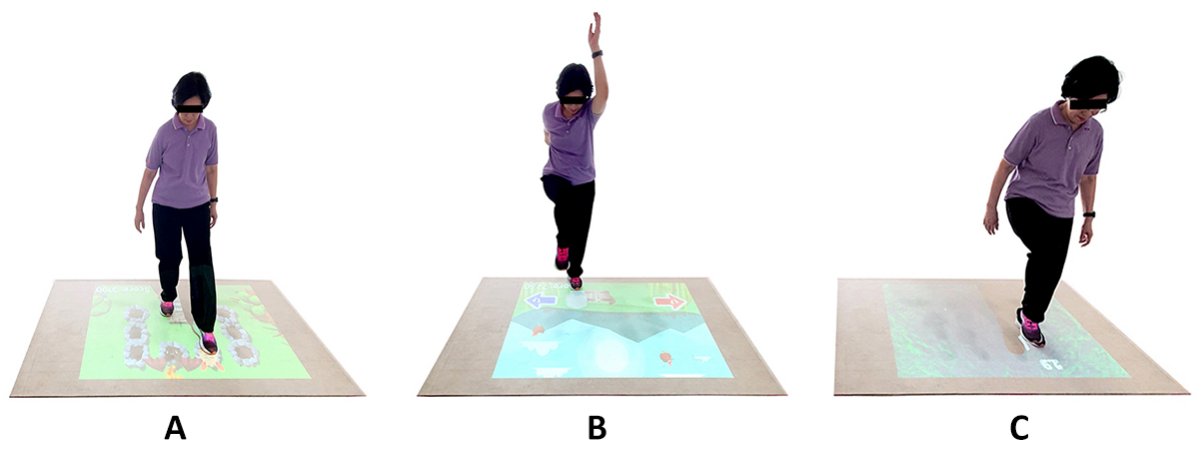
Examples of a participant playing the game-based exercise: (A) Whack a Mole, (B) Sky Falls, and (C) Crossing Poison River.

The framework of the game-based exercise comprised 6 components (Figure 3), including:

1. The Microsoft Kinect sensor: the depth sensor that was used to track and monitor full-body movements in 3D coordinates (ie, the x-, y-, and z-axes). The tracking data were then converted to the 24 points of the Joint ID Map (body skeleton model). In this study, 4 points of the Joint ID Map [43] (ANKLE_RIGHT, FOOT_RIGHT, ANKLE_LEFT and FOOT_LEFT) were used for the interaction between the user and the game.
2. Game programmers: the specialists who generated the digital game using the computer programming language and game engine software (the Unity 3D game engine software with Microsoft Kinect sensor V2 for Windows).
3. Domain knowledge: the experts having core knowledge and experience of physical and cognitive training programs for fall prevention in older adults.
4. Game-based training system: the digital game system, which comprised 5 games (Games I-V) with 3 levels (levels 1-3) and feedback (ie, score, response time, and error).
5. User and laptop computer: a system operator who was responsible for controlling the game-based training system while participants were playing the games.
6. Graphical user interface: a form of user interface that allows participants to interact with the game-based exercise (Figure 4). An example of a participant training with the game-based exercise prototype is displayed in Figure 5.

### Part II: Evaluation of the Target Users’ Experience

#### Participant Characteristics

A total of 5 community-dwelling older adults participated in the usability testing phase. Their mean age was 70.40 (SD 5.41) years (range 65-79 years). No participants had any experience with using exergames. They had low incidence rate of falls in the past 12 months, and they either did not take medication or took only one type. The participants’ characteristics are summarized in Table 2.

**Table 2:** Characteristics of the study participants.

Characteristic	Value	Median	Range
Age (years), mean (SD)	70.40 (5.41)	68	65-79
Height (cm), mean (SD)	155.20 (6.06)	156	149-164
Weight (kg), mean (SD)	53.40 (8.62)	55	39-60
BMI (kg/m^2^), mean (SD)	22.08 (2.63)	22.21	17.60-24.30
Education (years), mean (SD)	14.40 (5.90)	16	4-18
Types of medication, mean (SD)	0.20 (0.45)	0	0-1
Falls in the past year, n	1	0	0-1

#### User Experience in Using the Game-Based Exercise System

All 5 participants completed the game-based exercise and answered the PACES questionnaire. The average score on each item was between 6.66 and 7.00, which indicated greater levels of enjoyment. The 18-item PACES scores are illustrated in Table 3.

**Table 3:** Physical Activity Enjoyment Scale (PACES) rating scores (n=5). All items were rated on a 7-point scale from 1, strongly disagree, to 7, strongly agree.

Question	Participants						Response rating, mean (SD)
	S01	S02	S03	S04	S05		
1. I enjoy it; I hate it	7	6	7	7	6	6.80 (0.20)	
2. I feel interested; I feel bored	7	6	7	7	7	7.00 (0.00)	
3. I like it; I dislike it	7	7	7	7	7	7.00 (0.00)	
4. I find it pleasurable; I find it unpleasurable	7	7	7	6	6	6.66 (0.24)	
5. I am very absorbed in this activity; I am not at all absorbed in this activity	6	7	7	7	7	6.80 (0.20)	
6. It’s a lot of fun; it’s no fun at all	7	7	7	7	7	7.00 (0.00)	
7. I ﬁnd it energizing; I find it tiring	7	6	7	7	7	6.80 (0.20)	
8. It makes me happy; it makes me depressed	7	7	7	7	7	7.00 (0.00)	
9. It’s very pleasant; it’s very unpleasant	7	7	7	7	6	6.80 (0.20)	
10. I feel good physically while doing it; I feel bad physically while doing it	7	7	7	7	7	7.00 (0.00)	
11. It’s very invigorating; it’s not at all invigorating	7	7	7	6	7	6.80 (0.20)	
12. I am not at all frustrated by it; I am very frustrated by it	7	7	7	7	7	7.00 (0.00)	
13. It’s very gratifying; it’s not at all gratifying	7	7	7	6	6	6.66 (0.24)	
14. It’s very exhilarating; it’s not at all exhilarating	7	7	7	7	6	6.80 (0.20)	
15. It’s very stimulating; it’s not at all stimulating	7	7	7	6	7	6.80 (0.20)	
16. It give me a strong sense of accomplishment; it does not give me any sense of accomplishment	7	7	7	6	7	6.80 (0.20)	
17. It’s very refreshing; it’s not at all refreshing	7	7	6	6	7	6.66 (0.24)	
18. I felt as though there was nothing else I would rather be doing; I felt as though I would rather be doing something else	7	7	7	7	6	6.80 (0.20)	
Rating scale of all items (total points: 126)	125	123	125	120	120	123.00 (1.26)	

#### User Feedback and Suggestions

The feedback and suggestions provided by the users are presented in Table 4.

**Table 4:** Feedback and suggestions provided by the study participants for the game prototype.

Type of feedback or suggestion		Feedback
**Feedback**		
	Positive	“The games’ feature and appearance were very attractive and enhanced my motivation to complete the games.” “The games provided my performance with visible outcomes and scores which motivated me to try harder to get a better score in the next trial.” “The difficultly of each game was optimal; it was not too easy and not too difficult.” “The games had variety of forms and rules that challenged my physical and cognitive abilities.” “The games had a meaningful sound effect which helped me to identify my right or wrong responses.” “The games’ systems were quite simple to set up and easy to manage, thus it appeared to be feasible to use in the community or home settings.”
	Negative	“In the Whack a Mole, sometimes I did not step on the bomb, but it eventually blew up.” “In the Sky Falls, sometimes it was quite hard to control the movement of the bamboo basket even though I tried to alternate my stepping rhythmically.”
**Suggestions**		
	Game rules	Clearly state the game instructions and rules at the beginning
	Level design	Reduce the speed of dropping objects in the beginner level of Sky Falls Use different types of animals and vegetables for each difficulty level of Where Am I?
	Graphics/look and feel	Adjust the distance of each presented object in Whack a Mole
	Audio	Increase the display volume Use different background music for each game

## Discussion

### Principal Findings

In this study, we aimed to develop and test the usability of a virtual, interactive game-based training system that is focused on simultaneously training the physical and cognitive function (dual-tasking) of community-dwelling older adults. The core games were formulated by integrating the principal knowledge and existing evidence from the literature related to effective fall prevention exercise programs for older adults as well as by subjecting the games to critical appraisal from experts. We also assessed older adults’ experiences in terms of enjoyment and game features using the PACES questionnaire and a structured interview.

Several intervention studies have reported high dropout rates and limited use of technology-supported platforms for delivering exercise training programs [76-78]. Interactive game-based training may be considered as a more efficient approach for empowering user engagement, which contributes to positive outcomes of an intervention. However, many older adults tend to be less engaged with modern digital technology, and not all are accepting of it. To overcome this limitation, the UCD approach, which incorporates game design principles (ie, goals, rules, feedback, points, time, reward structures, levels, and aesthetics), was used in the process of designing and developing a game-based exercise prototype with an aim to motivate and engage older adults in exercising [54-60]. Researchers have identified the potential benefits of using the UCD concept for developing exergames for older adults with and without health-related problems. For example, Hemingway et al [53] used the UCD process to develop a mobile game to influence the behavior of HIV service uptake among a key population. Lange et al [27] established an interactive game-based program constructed on a UCD design process for training the dynamic balance of individuals who have previously experienced a stroke. Howes et al [79] also used UCD to develop the bespoke Active Computer Gaming system to deliver strength and balance exercise programs for older adults. Together, the present and previous findings consistently suggest that the UCD approach is a core process that should be embedded in health games to ensure usability and acceptability. Therefore, the target users may benefit fully from exergames technology.

To our knowledge, our game-based exercise is the first game prototype that was mainly designed to support older adults in combined physical-cognitive exercising using the Microsoft Kinect motion sensor. In particular, the game-based exercise focused on the core impairment aspects that are related to falls in older adults, including balance and stepping performance as well as executive function, attention, and memory. Several investigators have consistently reported that the most important component of exercise programs for fall prevention is balance training [6,7]. In addition, stepping training, a form of highly specific balance training, has shown to be an effective fall prevention strategy [80]. Thus, balance and stepping training were included in the game-based exercise. Regarding cognition, declined executive function, attention, and memory have been identified as crucial contributors to falls [11-13,19]. Thus, adding these cognitive components to physical training may potentially enhance the efficacy of fall prevention programs for older adults. In addition, age-related perception and sensation decline in older adults were considered. Therefore, visual and audio presentations, such as the size and distance of target objects as well as the level of sound volume, were included. Findings from the study demonstrated that the target users viewed the game-based exercise prototype as an enjoyable and practical intervention approach for their physical and cognitive training at home and in community settings. This may be because the development of the game-based exercise prototype incorporated the current knowledge regarding the key contributing factors for training continuation by using exergames among older adults. These factors gradually increase the level of game difficulty, provide clearer feedback, and offer a simple setup [81]. In this way, we expected that the newly developed exergames would overcome the barrier to exercise in older adults. This enjoyment (determined by PACES scores) and positive feedback from the users may be, at least in part, due to the fundamental elements of the game-based exercise, which feature a real-time interface display and feedback. Consistent with previous studies, our games system provides feedback, including scores and performance outcomes (ie, response time, error), which enhances the motivation of the users [82-84]. Moreover, the positive responses from older learners who are unfamiliar with new technologies can be attributed to the design features of the game-based exercise prototype, such as having a user-friendly interface and providing optimal levels of task difficulty [85]. Nevertheless, some comments indicated that further refinements are required prior to implementation of the game-based exercise among older adults in a realistic context.

### Limitations

This study has certain limitations that need to be acknowledged. This study is a preliminary study that involved a small number of participants who were all female. Further, results on the enjoyment and experiences of using the game-based exercise prototype were obtained from a single training session. Thus, the findings should be considered preliminary and interpreted with caution. Future studies with larger sample sizes, a balanced gender ratio, and data obtained from multiple training sessions are warranted. Another limitation concerns the hardware specification of the Microsoft Kinect sensor. In this study, the capture volume of the Kinect sensor was restricted to 0.5 to 4.5 m, which partly limited the design of the configurations of the games. Further studies should consider using multiple Kinect sensors to cover a greater capture volume. Moreover, our game system was designed for the individual player. Exergames systems that allow group players should be considered for promoting social interaction. Finally, this study investigated the enjoyment during game engagement using the 18-item PACES questionnaire. In future studies, the 8-item version of PACES would be an appropriate questionnaire to reduce the completion time.

### Conclusions

This preliminary study demonstrated a prototype of a game-based exercise for older adults using the Microsoft Kinect sensor. The game-based exercise prototype contained combined physical and cognitive training elements with different levels of difficulty. The developed game-based exercise was well accepted by the target users, with prominent enjoyment and positive feedback. Thus, the game-based exercise appears to be a promising tool for enhancing older adults’ motivation to engage in physical-cognitive exercise with the aim to reduce the risk of falls.

## Abbreviations

PACES: Physical Activity Enjoyment Scale
UCD: user-centered design
